# Commentary: An enzymatic pathway in the human gut microbiome that converts A to universal O type blood

**DOI:** 10.3389/fimmu.2020.00772

**Published:** 2020-04-28

**Authors:** Oloche Owoicho, Charles Ochieng' Olwal, Osbourne Quaye

**Affiliations:** Department of Biochemistry, Cell and Molecular Biology, West African Centre for Cell Biology of Infectious Pathogens, University of Ghana, Accra, Ghana

**Keywords:** blood transfusion, HLA-I, platelet transfusion, thrombocytopenia, platelet transfusion refractoriness, epithelial cells

In blood transfusion, matching of blood types is an essential requirement since plasma of individuals of blood group A contains antibodies against blood group B and vice versa, making the two blood groups incompatible. The incompatible transfusion can result in red blood cell (RBC) lysis, and lead to death. In contrast, blood group O type RBCs can be transfused universally to patients of the same rhesus type. Therefore, there is need for adequate supply of blood group O RBCs to meet the demands of blood transfusion. To reduce the blood transfusion challenge, Rahfeld et al. (1) characterized an A-O ABO blood group antigen-converting enzyme in human gut microbiome. These human gut microbiota produce galactosaminidase, which converts blood group A to O of the same rhesus type and therefore have the potential to significantly reduce the incompatibility challenge of blood transfusion. These findings by Rahfeld et al., apart from accelerating the progress toward universal RBC, provides a rational approach for management of thrombocytopenia. Thrombocytopenia is a hematological disorder characterized by spontaneous and/or abnormally prolonged bleeding events (2). Clinical management of thrombocytopenia involves platelet transfusion (3). In persistently thrombocytopenic patients, multiple platelet transfusion is required. However, a significant number of these patients develop platelet transfusion refractoriness (PTR), which is repeated failure to meet the desired level of blood platelets after platelet transfusion (4). Although the presence of antibodies against donor human platelet antigens have been reported in some PTR cases, alloimmunization against donor classical human leukocyte antigen class I (HLA-I) has been the major immune cause of PTR (5).

HLA-I are found in all nucleated cells and platelets, and present intracellular pathogens and malignant cell-derived antigenic peptides to cytotoxic T lymphocytes (CTLs) for immune surveillance. HLA-I are heterodimers comprised of a heavy α-chain encoded by *HLA-A, HLA-B* or *HLA-C*, and a soluble β2-microglobulin (β2m) encoded by *B2M* (6). Considering that the *HLA-A, HLA-B*, and *HLA-C* are some of the most polymorphic genes in human (7), getting compatible donor-recipient HLA-I allotypes will be a heinous task.

Although studies have suggested approaches that could surmount PTR due to HLA-I alloimmunization, none of these are clinically viable alternatives for HLA-I-matched platelets transfusion. A study suggested that eculizumab could confer protection against PTR but the potential of the drug has not been validated (4), and the immune system suppression by the drug could increase the risk of contracting opportunistic infections (8). Other studies have explored CRISPR/Cas9 technology to obtain HLA-I knockout platelets, which are functionally comparable to platelets circulating in human blood (5, 9), but the prospects of transfusing these engineered universal platelets into humans are likely to be fettered by ethico-legal concerns. Moreover, the current CRISPR/Cas9-mediated generation of universal platelets involves the use of viral vectors, which may be oncogenic. Even though studies have used acids to strip off platelets from HLA-I molecules (10, 11), the method may cause some loss of platelets (12).

We envisage that the findings by Rahfeld et al. herald a paradigm shift in transfusion science, and propose the possibility of screening the human gut microbiome for an HLA-I degrading enzyme (HLA-I-ase), which can be utilized to generate mutation-free HLA-I-deficient universal platelets. Subsequently, we share how this could be achieved, and the possible application in transfusion medicine.

## Rational Approach to HLA-I-ase Prospecting

Metagenomic libraries of all microorganisms present in a sampled environment could be created and screened to identify the enzymes encoded by the metagenome (1). A potential source of HLA-I-ase producing microorganisms should have abundant population of HLA-I-ase-expressing microorganisms to increase the chances of finding relevant HLA-I-ase. Thus, an anatomical site such as the gastrointestinal mucosa, where microorganisms interact with epithelial cells, is naturally a potential source of HLA-I-ase. Stool samples from healthy individuals could be pooled and used for the metagenomics libraries for HLA-I-ase encoded genes as has been done for ABO blood antigens degrading enzymes (1).

## Choosing the Right HLA-I-ase: What to Consider

Due to the highly polymorphic nature of HLA-I gene, an ideal HLA-I-ase should be specific for HLA-I expressed on cell surfaces and have a robust enzymatic capacity to degrade a wide array of HLA-I allotypes. The ideal enzyme should degrade HLA-I in such a manner that it prevents anti-HLA-I antibodies in alloimmunized patients from binding platelets and forestalls phagocytosis of transfused HLA-I incompatible platelets. The enzyme will likely degrade the α_3_ domain of HLA-I, which provides the binding site for CD8 co-receptor of CTLs, or the β2m, which provides stability for HLA-I molecule. Degrading the α_3_ domain and/or the β2m regions will destabilize HLA-I from the cell membrane and render it inaccessible to anti-HLA-I antibodies.

## Generation of Universal Platelets Using HLA-I-ase

Scalable production of functionally viable and transfusable platelets using human induced pluripotent stem cells (iPSCs) as replenishable source of megakaryocytes has been established (12). What is yet to be achieved is development of mutation-independent universal platelets that overcomes alloimmunization against HLA-I and enhances the management of PTR. Subjecting iPSCs-derived platelets to HLA-I-ase in optimal conditions will render the cells HLA-I surface free. These universal cells can then be purified and passed through quality checks to confirm their universal application. The quality check should include the interrogation of enzyme-treated cells for the presence of HLA-I on the cell surface. HLA-I molecules have been assayed using flow cytometry (6), hence the method could be explored for this purpose. Incorporating HLA-I-ase treatment to the established scalable production of megakaryocytes from iPSCs will ensure sufficient supply of universal platelets, obviate HLA-I pre-transfusion typing, reduce platelet transfusion cost, and incidence of PTR.

## Conclusions

Elucidation of HLA-I-ase will revolutionize blood transfusion medicine, especially the management of thrombocytopenia and PTR. Furthermore, the concept hold promise for transplantation science as the use of the enzyme could be extended to other cell types, tissues or organs to minimize or even eliminate graft versus host rejection. A potential limitation of this approach, which the scientific community may have to grapple with, is that nucleated cells in tissues and organs could replace the enzymatically cleaved HLA-I. Nevertheless, the search for an effective HLA-I-ase is warranted to avert PTR.

## Author Contributions

OO and CO conceived and wrote the first draft of the manuscript. OQ critically reviewed the manuscript. All the authors finalized and approved the manuscript.

## Conflict of Interest

The authors declare that the research was conducted in the absence of any commercial or financial relationships that could be construed as a potential conflict of interest.
